# The Hidden Burden of Hemifacial Spasm: A Systematic Review of Non‐Motor Symptoms

**DOI:** 10.1002/mdc3.70574

**Published:** 2026-03-05

**Authors:** Miriam Carvalho Soares, Jacy Bezerra Parmera, Pedro Augusto Sampaio Rocha‐Filho

**Affiliations:** ^1^ Neuropsychiatry and Behavioral Sciences Federal University of Pernambuco (UFPE) Recife Brazil; ^2^ Department of Neurology, Hospital das Clínicas Faculdade de Medicina da Universidade de São Paulo (HC‐FMUSP) São Paulo Brazil; ^3^ Division of Neuropsychiatry, Centro de Ciências Médicas Universidade Federal de Pernambuco (UFPE) Recife Brazil

**Keywords:** depression, facial pain, headache, hemifacial spasm, insomnia

## Abstract

**Background:**

Hemifacial spasm (HFS) is a chronic neurological disorder characterized by involuntary contractions of facial muscles. Traditionally regarded as a motor condition, HFS encompasses a spectrum of non‐motor symptoms that are often overlooked but significantly affect patients’ quality of life.

**Objectives:**

This systematic review aimed to synthesize current evidence on the prevalence and clinical relevance of non‐motor manifestations in HFS.

**Methods:**

A systematic search was conducted across PubMed, Cochrane, and EMBASE databases up to June 29, 2025, following PRISMA guidelines. Eligible studies included cross‐sectional, cohort, case–control, and case series designs reporting pain, psychiatric symptoms, or sleep disturbances in HFS. Risk of bias was assessed using the Joanna Briggs Institute critical appraisal tools according to study design.

**Results:**

Twenty‐two studies met inclusion criteria. Headache was reported in varying frequencies, ranging from 24.2% to 60% of cases, and facial pain in 33.3%, both frequently improving after botulinum toxin (BoNT) therapy. Psychiatric comorbidities were prevalent, with depression and anxiety reported in 5.7% to 29% and 9.1% to 19.5% of patients, respectively. Sleep disorders, mainly insomnia, occurred up to three times more often in HFS patients than in controls. BoNT therapy demonstrated beneficial effects beyond motor control, reducing pain, anxiety, and depressive symptoms.

**Conclusion:**

HFS is a multidimensional disorder with substantial non‐motor burden. Recognition and management of pain, psychiatric, and sleep‐related symptoms are essential to improving treatment outcomes and overall patient well‐being.

Hemifacial spasm (HFS) is characterized by involuntary contractions of the muscles innervated by the facial nerve, typically affecting only one side of the face. It is a type of peripheral myoclonus and is classified into primary and secondary forms. Primary HFS is usually caused by compression of the facial nerve at its root entry/exit zone near the pons in the brainstem, often by nearby blood vessels. Secondary HFS is associated with structural damage to the facial nerve, resulting from conditions such as tumors, demyelinating diseases, infections, or trauma.^1^

The global prevalence of HFS is estimated at around 11 cases per 100,000 people. It is most commonly diagnosed between the fourth and sixth decades of life and affects women twice as often as men.^2^ Beyond motor symptoms, the condition significantly affects quality of life, impacting social interactions, vision, and speech.^1^ Non‐motor symptoms and comorbidities, such as pain, psychiatric issues, and sleep disorders in HFS, are frequently overlooked during clinical evaluations, mainly because the focus tends to be on managing the prominent motor symptoms, which are often promptly addressed and also concentrate research focus.^3^ However, non‐motor symptoms are also prevalent and can substantially influence patients’ well‐being.

Treatment options for HFS range widely from simple non‐pharmacological measures, such as applying warm compresses, to more invasive interventions like microvascular decompression (MVD) surgery, which remains the current gold‐standard treatment.^4^ Due to its invasive nature, MVD carries notable risks, including hearing loss, facial weakness and intracranial hemorrhage.^5^

As a result, clinical practice has increasingly shifted away from surgery toward the use of botulinum toxin type A (BoNT).^6^ Additionally, BoNT may also alleviate non‐motor symptoms either directly by inhibiting the release of pain‐related neuropeptides or indirectly by reducing muscle contractions and associated discomfort.^7^, ^8^

Non‐motor symptoms can also influence motor outcomes. Studies have shown that improvements in motor symptoms do not always align with patients’ subjective sense of well‐being.^9^ Psychological factors, including mood and self‐perception, can significantly influence how patients experience their condition, a phenomenon that is evident not only in clinical research but also in everyday practice. Therefore, effective management requires both objective measures and attention to subjective experiences.

This review aims to explore the prevalence, types, and impact of non‐motor symptoms and comorbidities in patients with HFS providing a synthesized overview of the current evidence to support practical care approaches. This review focused on pain‐related symptoms, depression, anxiety and sleep disturbances, as these domains are among the most frequently reported non‐motor manifestations of HFS and have been more consistently evaluated using structured instruments. Other non‐motor features, including fatigue, cognitive complaints and autonomic symptoms, were screened but were not included in the primary synthesis due to sparse, heterogeneous, and predominantly descriptive reporting.

Given the distinct etiological and clinical profiles of secondary HFS, the present review was restricted to primary HFS, with secondary causes intentionally excluded to enhance interpretability and generalizability within this population.

## Methods

The current review was conducted in accordance with the Preferred Reporting Items for Systematic Reviews and Meta‐Analyses (PRISMA) guidelines.^10^ The review was registered with the International Prospective Register of Systematic Reviews (PROSPERO, ID: CRD420250653777). Ethical committee approval was not required, since no primary data sources were included.

### Inclusion and Exclusion Criteria

This review included cross‐sectional studies, cohort studies, case–control studies, case reports and case series. Only articles published in the English language were included. The selected articles should focus on outcomes such as epidemiological descriptions and clinical characterization of the specific non‐motor symptoms.

Exclusion criteria were: articles which focused on procedures and surgical techniques outcomes and on secondary cranial neuralgias or neuropathies (eg, trigeminal, occipital); articles about secondary hemifacial spasms (eg, cases resulting from structural lesions such as tumors, aneurysms or after facial nerve paralysis); articles about pain after surgical procedures and hemifacial spasm following facial paralysis. Only studies of primary HFS were included, with secondary forms excluded to reduce etiological heterogeneity and ensure clinical and pathophysiological comparability.

### Search Strategy, Identification of Relevant Studies and Data Extraction

Three databases were searched: PubMed/MEDLINE, Cochrane and EMBASE databases. The search used the following combination of descriptors/MeSH terms: (“hemifacial spasm” AND “headache”), (“hemifacial spasm” AND “facial pain”), (“hemifacial spasm” AND “pain”), (“hemifacial spasm” AND “anxiety”), (“hemifacial spasm” AND “depression”), (“hemifacial spasm” AND “insomnia”), and (“hemifacial spasm” AND “sleep”). Literature search was conducted independently by two researchers (MCS and JBP) up to June 29, 2025. In addition, reference lists of key studies were manually screened.

Title and abstract screening were conducted by the two researchers (MCS and JBP) using Rayyan (https://www.rayyan.ai), a web‐based tool designed to facilitate efficient screening in systematic reviews.^11^ Disagreements between reviewers were resolved by consensus. In cases of disagreement, a senior researcher (PASRF) reviewed the article to make the final determination regarding inclusion or exclusion.

Data were extracted by focusing on information such as the author's name, study objective, year of publication, language, study type, mean age and standard deviation (SD) of the participants, non‐motor symptoms associated with hemifacial spasm, including headache, facial pain, anxiety, depression and sleep disturbances. Both data extraction and risk of bias assessment were performed independently.

### Risk of Bias and Quality of Evidence

The risk of bias was assessed using the critical appraisal tools developed by the Joanna Briggs Institute (JBI),^12^ selected according to the specific study design of each included article. Each study design was assessed using the corresponding JBI critical appraisal tool: cohort studies with the Cohort Checklist, case‐control studies with the Case‐Control Checklist, analytical cross‐sectional studies with the Cross‐Sectional Checklist, case reports with the Case Report Checklist, and case series with the Case Series Checklist.^13^ All articles were independently assessed by two reviewers (MCS and JBP).

Risk of bias was assessed using design‐specific JBI critical appraisal tools. It is important to considerate that these instruments evaluate internal methodological quality within each study design rather than allowing direct comparison across different designs. Consequently, a classification of low risk of bias reflects adherence to methodological standards appropriate for that design and does not imply a high overall level of evidence, particularly for inherently descriptive designs such as case reports or uncontrolled observational studies.

### Synthesis

A systematic, narrative synthesis was conducted in this review, as a quantitative meta‐analysis was not feasible due to significant heterogeneity in study designs and outcome measures. To ensure clarity, results and data extraction were organized by symptom category. Data were grouped by population characteristics (sample size, age, gender) and by the type of non‐motor symptoms, including the tools used for assessment and reported prevalence. When available, information from complementary exams was also included.

## Results

A total of 1279 articles were initially identified, of which 982 were excluded following the duplicate analysis. Of the 297 screened, 245 were excluded (65 articles were excluded due to lack of clinical characterization/description; four articles for addressing patients with spasm following facial nerve paralysis; 122 articles focused only on surgical procedure outcomes; 49 papers addressed spasms arising in patients with structural lesions; and five articles discussed facial pain in the context of bruxism/temporomandibular joint dysfunction). Of the 52 articles selected for full‐text review based on the eligibility criteria, 30 were excluded because they lacked clear descriptions of symptoms, did not specify the instruments used for assessment, or provided insufficient patient characterization.

**Figure 1:**
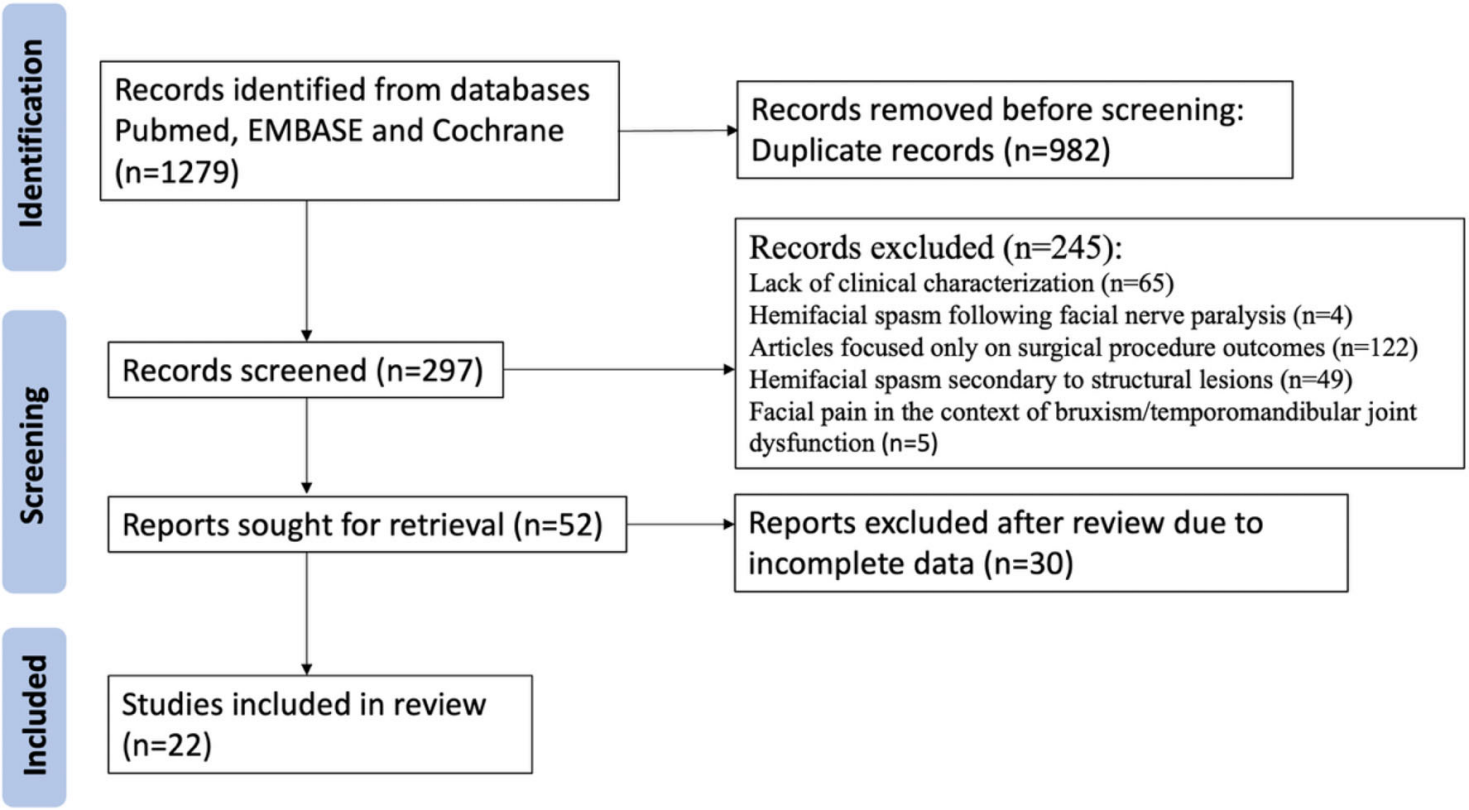
PRISMA flowchart detailing the selection of studies included the systematic review.

Twenty‐two articles were included: eight case reports, seven cross‐sectional studies, two case–control studies, four cohort studies, and one case series. The study selection process is summarized in the PRISMA flow diagram (Fig. 1).

Most studies (n = 19/22, 86%) were judged to have a low risk of bias, with well‐defined inclusion criteria, appropriate measurement tools, valid outcome assessments, and adequate statistical analyses (supplemental Table S1).^14^ A smaller proportion (n = 3/22, 14%) showed a moderate risk of bias, mainly due to design‐related limitations such as insufficient descriptions of group characteristics or inadequate reporting of adverse events in case reports. Notably, no study was excluded from the synthesis due to methodological flaws. Overall, the evidence quality was deemed acceptable, despite the considerable heterogeneity in study designs.

### Headache and Facial Pain

Eleven studies addressing headache and facial pain in patients with HFS were included in this review. Table 1 summarizes these studies. The association between pain and HFS has been only sparsely documented, with reported headache prevalence among patients with HFS ranging from 24.2% to 60% across studies, and facial pain reported in approximately 33% of cases.^15^, ^16^

**TABLE 1:** Headache and facial pain in individuals with hemifacial spasm (HFS)

Author	Year	Number of patients (*n*)/sex	Study type	Results	Neuroimage
Harrison et al^16^	2008	n = 33 Age: 69.8 (41–90) years. Sex: not mentioned	Retrospective cohort	Among 33 BoNT‐treated HFS patients, headache occurred in 8/33 (24%) and facial pain in 11/33 (33%). Half of those with headache improved (mean relief 3.7 ± 1.1), while 82.8% with facial pain reported benefit.	Not mentioned
Mizuma et al^15^	2017	n = 51 Age: 64 ± 14 years. Female: 41 (80.4%)	Retrospective cohort	51 HFS patients were assessed before and after BoNT treatment; 17 (33%) reported worsening or new onset of headache (especially TTH) associated with HFS. 12/17 patients (70.6%) reported improvement of headache after BoNT therapy.	Not mentioned
Peeraully et al^18^	2013	n = 70 Age: 58 ± 13 years. Female: 47 (67.1%)	Cross‐sectional	Among 70 HFS patients, 60% had headaches, and in 20 cases headaches were triggered or worsened by HFS. Headache was associated with greater spasm severity (*P* < 0.001), and BoNT treatment significantly improved HFS‐related headaches (*P* < 0.001).	Not mentioned
Barahona‐Hernando et al^23^	2012	n = 3 Age: 31, 31 and 36 years Female: 1	Case series	Spasms consistently appeared after pain onset, with migraine preceding HFS by 3–8 years and HFS emerging 3–4 years later (one simultaneous case). Spasms aligned temporally with migraine attacks, and one patient improved with topiramate 100 mg.	MRI/MRA was normal in one case and showed vascular contact with the facial nerve in two cases (AICA identified in one).
Iida et al^19^	2004	n = 1 Age: 65 years. Male	Case report	Left periorbital aching pain for 4 years and left HFS that persisted throughout the same time. Pain intensity responded to BoNT (improvement of facial pain from 9 to 2 on VAS). Sustained pain relief was observed after MVD of the seventh cranial nerve.	Angiogram revealed a loop of the left posterior inferior cerebellar artery compressing the seventh nerve.
Husid et al^20^	2005	n = 1 Age: 36 years. Female	Case report	Chronic right‐sided migraine without aura was accompanied by ipsilateral HFS. Spasms occurred exclusively during headache attacks and resolved fully with headache relief. No features of functional movement disorder were present, and no treatment details were reported.	Not mentioned
Nakazato et al^21^	2006	n = 1 Age: 34 years. Male	Case report	Left cluster headache with secondary left‐sided HFS, emerging 2 years later; spasms intensified with headache severity and resolved with oxygen or sumatriptan, disappearing spontaneously after 4 months.	MRI and MRA did not show any abnormality.
Cuadrado et al^22^	2006	n = 1 Age: 35 years. Female	Case report	A 17‐year history of strictly left‐sided migraine was accompanied by ipsilateral HFS that appeared at peak headache intensity, lasted 30–60 minutes from migraine onset, and subsided as the headache improved.	MRI and MRA were normal.
Alonso‐Navarro et al^24^	2007	n = 1 Age: 52 years Female	Case report	Right‐sided migraine without aura developed at age 21, 2 years after the onset of right‐sided HFS. Both conditions improved completely with topiramate 75 mg/day, worsened after discontinuation, and improved again when treatment was restarted.	Not mentioned
Fenech et al^41^	2017	n = 1 Age: 57 years. Male	Case report	Sequential bilateral trigeminal neuralgia and HFS developed over two decades, with good outcomes after bilateral MVD. Persistent right‐sided HFS remains well controlled with botulinum toxin.	MRI showed bilateral trigeminal and facial nerve compression by the SCA and AICA, respectively.
Punj et al^42^	2023	n = 1 Age: 54 years. Male	Case report	Left HFS at 44 was followed by right‐sided trigeminal autonomic cephalalgia and occipital neuralgia at 49, with complete remission after combined nerve blocks, botulinum toxin, pulsed radiofrequency, and indomethacin.	Not performed (patient refused).

Abbreviations: AICA: anterior inferior cerebellar arteries; BEB: blepharospasm; BoNT: Botulinum toxin; MRA: magnetic resonance angiography; MRI: magnetic resonance imaging; MVD: microvascular decompression; SCA: superior cerebellar arteries; TTH: tension‐type headache; VAS, visual analog scale.

Evidence on the association between HFS and headache is derived primarily from observational cohort and cross‐sectional studies, with case reports providing supportive, illustrative data. Among the higher‐quality observational evidence, a retrospective cohort study of 51 HFS patients conducted at a tertiary center in Japan, analyzed medical records of HFS patients at baseline and 6 weeks after BoNT injection. They found that tension‐type headache (TTH), as defined by the International Classification of Headache Disorders, 3rd edition (ICHD‐III beta)^17^ coexists with HFS in up to 33% of cases. Moreover, in their study, headache intensity significantly decreased following BoNT injections. Pain intensity scores (measured by a numerical rating scale) improved from 7 (5–9) to 0 (0–5) (*P* < 0.01), and HIT‐6 (Headache Impact Test) scores improved from 55 to 44 (*P* < 0.001). Logistic regression analysis revealed that the worsening/new onset of TTH was associated with the presence of stress (OR: 43.11; 95% CI: 2.95–629.39; *P* < 0.001) and a previous history of chronic headache (at least 15 days/month of pain, for over 3 months) (OR:28.53; 95% CI: 2.96–275.10; *P* < 0.001).^15^

Peeraully et al conducted a cross‐sectional study involving 70 patients with HFS in Singapore. The authors reported that 60% of individuals with HFS experienced headache, and in 28% of cases, the onset or worsening of headache were precipitated by HFS symptoms, a subgroup designated as “HFS‐related headache.” Among these 20 participants with HFS‐related headache, 13 developed them after the onset of HFS, whereas seven had pre‐existing headaches that subsequently worsened. The study applied a structured questionnaire encompassing demographic data, HFS clinical features (duration, frequency, and severity graded on a 0–4 scale), and two validated instruments: the HFS‐7 quality‐of‐life scale for HFS symptoms and the HIT‐6 score for headache impact measurement. Patients with HFS‐related headache demonstrated higher HFS motor severity and HIT‐6 scores, indicating greater motor symptom intensity and headache impact compared to those with headaches unrelated to HFS. In contrast, there was no significant difference in HFS‐7 quality‐of‐life scores between groups (mean = 5 vs 4; *P* = 0.074). Headache characteristics also differed: those with HFS‐related headaches were more likely to describe the pain as tight in nature (60% vs 29.6%). Notably, therapeutic response to BoNT injections was strikingly distinct. Improvement occurred in 75% of patients with HFS‐related headaches, while none of those with unrelated headaches reported benefit (*P* < 0.001). In logistic regression analysis, HFS severity emerged as the only independent predictor of HFS‐related headache (*P* = 0.006; OR 19.1; 95% CI 2.35–155.64).^18^

Harrison et al. conducted a retrospective cohort study to investigate the efficacy of BoNT injections in the relief of headache and facial pain among patients with HFS and blepharospasm. The study included 85 individuals, of whom 33 had HFS. Among those with HFS, the prevalence of headache was 24% and of facial pain, 33%, with the latter predominantly localized to the periorbital region. Following treatment with BoNT, 50% of patients with headache reported improvement, with a mean improvement score of 3.7 ± 1.1 on a five‐point scale (0 = no relief and 5 = total relief). Likewise, 82.8% of those with facial pain described symptomatic benefit, with a mean improvement degree of 4.3 ± 0.9.^16^

Evidence from case reports and small case series, while limited by selection bias and lack of comparators, offers mechanistic and temporal insights. Four case reports described the coexistence of HFS and headache. Iida et al described a 65‐year‐old man experienced left periorbital pain concurrent with ipsilateral HFS for 4 years, with marked pain relief following botulinum toxin therapy and sustained remission after microvascular decompression of the facial nerve, which was compressed by a posterior inferior cerebellar artery loop.^19^ Husid et al reported a 36‐year‐old woman with right‐sided chronic migraine in whom facial spasms appeared exclusively during headache attacks, resolving completely with pain relief.^20^ Similarly, Nakazato et al described a 34‐year‐old man with cluster headache and ipsilateral HFS which intensified with headache severity and improved with acute headache treatment.^21^ Finally, Cuadrado et al detailed a 35‐year‐old woman with long‐standing unilateral migraine in whom HFS episodes occurred only during headache peaks and subsided as pain diminished.^22^

Additionally, two studies described cases of HFS and migraine co‐occurrence with significant symptom improvement following topiramate treatment for migraine. It has been suggested that abnormal transmission along the facial nerve could contribute to overlapping symptoms and that topiramate may help modulate this abnormal neural activity.^23^, ^24^

### Psychiatric Symptoms

Eight studies addressing psychiatric symptoms and psychiatric disorders in patients with HFS were included in this review. Table 2 summarizes these studies.

**TABLE 2:** Frequency of psychiatric symptoms and psychiatric disorders in hemifacial spasm (HFS) patients

Author	Year	Number of patients (n)/Age/Sex	Study type	Main results
Yuksel et al^31^	2017	n = 40 Age: 75% (30) were older than 50 years Female: 21 (52.5%)	Prospective cohort	The study assessed stigma in HFS patients and examined the effects of BoNT on depression in a Turkish cohort. BDI, SF‐36 and HFS‐7 were used to assess the depressive symptoms, quality of life and HFS impact in daily life, respectively. BoNT treatment reduced depressive symptoms, with mean BDI scores decreasing from 9.28 to 6.7 (*P* < 0.01) and moderate depression falling from 17.5% to 12.5%. No significant changes were observed in HFS‐7 or SF‐36 scores.
Wang et al^39^	2022	n = 95 Age: 54.7 ± 11.8 years Female: 50 (52.6%)	Prospective cohort	The study evaluated changes in mental health after BoNT treatment in HFS patients and compared them with healthy controls. HFS patients showed higher baseline SCL‐90R scores for anxiety, depression, somatization, and phobia than healthy controls. After BoNT, psychological distress improved significantly (61.9% to 35.7%; *P* = 0.03).
Tan et al^26^	2006	90 HFS patients and 96 healthy controls Age: Patients: 55 ± 12 Controls: 51 ± 15 years. Female: HFS: 54 (60%) Control: 53 (55%)	Case–control	To use the SCL‐90R to evaluate symptoms across nine psychological domains in patients with HFS compared to healthy controls. HFS patients had higher scores of generalized anxiety (*P* = 0.0001), phobic anxiety (*P* = 0.005) and depression (*P* = 0.03) in the SCL‐90R than matched controls. A greater prevalence of anxiety disorder (19.5%) in HFS compared to controls (3.8%) was also found (*P* < 0.001), based on DSM IV criteria.
Tan et al^25^	2005	90 HFS patients Age: 54.4 ± 11.1 Female: 5 (58.9%)	Cross‐sectional	Prevalence of depressive disorder was 16.7%. Female gender and younger age were risk factors (*P* = 0.07). Severity of HFS independently predicted BDI scores (*P* < 0.0001).
Dias et al^27^	2010	29 HFS patients Age: 60.5 ± 11.7 years Female: 22 (75.8%)	Cross‐sectional	Prevalence and severity of psychiatric disorders were evaluated in blepharospasm vs. HFS patients, using DSM‐IV–based instruments. Frequency of depressive disorder in HFS patients was 13.7%; dysthymia: 10.3%; social phobia: 24.1%; generalized anxiety disorder: 10.3% and suicidal ideation was 13.7%. Diagnoses were made based on DSM IV.
Fontenelle et al^28^	2011	n = 31 Age: 65.8 ± 11.9 years Female: 21 (67.7%)	Cross‐sectional	The study compared the prevalence and severity of obsessive‐compulsive symptoms in patients with blepharospasm vs. HFS, using OCI‐R, BDI and BAI. Major depressive disorder prevalence in HFS patients was 29%, generalized anxiety disorder was 12.9%, hypomania 3.2%, panic disorder: 3.2%, agoraphobia: 6.4%, based on DSM IV.
Lin et al^43^	2014	n = 1003 Age: 46.6 ± 11.5 years. Female: 643 (64.1%)	Cross‐sectional	1003 HFS Chinese HFS patients were interviewed. 91 (9.1%) were diagnosed with depression and 57 (5.7%) with anxiety, as assessed by the SDS and SAS, respectively.
Cai et al^30^	2024	n = 151 Age: 56.85 ± 10.15 years Female: 104 (68.4%)	Cross‐sectional	The study evaluated how body‐image disturbances relate to social anxiety in Chinese patients with HFS. High levels of social anxiety were found in 151 HFS patients, (mean SADS score: 19.07 ± 7.40; cutoff ≥12). Direct effects of body image on social anxiety in HFS patients were found.

Abbreviations: BAI, beck anxiety inventory; BDI, beck depression inventory; OCI‐R, obsessive‐compulsive inventory‐revised; SADS, social avoidance and distress scale; SAS, self‐rating anxiety scale; SCL‐90R, symptom checklist‐90R; SDS, self‐rating depression scale.

The prevalence of depressive disorders among HFS patients varies across studies. Tan et al in a cross‐sectional study reported a depression prevalence of 16.7% among patients with HFS based on DSM‐IV criteria, with younger women showing a higher risk. Additionally, HFS severity score positively correlated with depression (OR = 128.4, *P* = 0.001). However, duration of HFS was not different between depressed HFS patients and non‐depressed (*P* = 0.96). The Beck Depression Inventory (BDI) proved to be a useful screening tool in this context and female sex were found to be more at risk of depression (OR = 66.23, *P* = 0.009). Multivariate analysis identified sex, HFS severity, and the presence of other medical conditions (eg, Diabetes mellitus, hypertension, heart disease, hyperlipidemia, stroke, tumors, smoking or alcohol intake) as independent predictors of depression in patients with HFS.^25^

A subsequent case–control study by the same group confirmed significantly higher anxiety levels among patients with HFS. To ensure robust comparisons, the authors employed two distinct control groups: (1) healthy individuals matched for age, sex, and race, to determine baseline anxiety rates in the general population; and (2) outpatient controls attending the same clinic for unrelated conditions, to exclude the confounding effect of being in a medical environment or having a chronic illness. This dual‐control approach strengthened the attribution of anxiety specifically to HFS rather than to nonspecific clinical factors. Anxiety was assessed through two complementary instruments. The Symptom Checklist‐90R (SCL‐90R) provided a broad profile of psychological distress across nine domains, enabling differentiation between generalized psychopathology and anxiety‐specific symptoms. The Hamilton Anxiety Rating Scale (HAM‐A) was used to quantify anxiety severity and confirm its clinical relevance. Using these methods, anxiety prevalence reached 19.5% among HFS patients, compared with 3.8% in the second control group (*P* < 0.0001). HAM‐A scores were also higher in the HFS group (10.0 ± 8.0 vs. 5.0 ± 5.0, *P* = 0.004). Eight of 41 HFS patients (19.5%) had HAM‐A scores ≥18 (threshold for clinically significant anxiety) compared with 2 of 52 individuals in the second control group (3.8%; *P* = 0.02). All affected patients also met DSM‐IV criteria for generalized anxiety disorder on clinical interview.^26^

Dias et al., through a cross‐sectional study conducted in Brazil, observed a 13.7% prevalence of major depression, 13.7% of suicidal ideation, and 10.3% of generalized anxiety disorder among HFS patients, according to DSM‐IV diagnostic criteria. Additionally, 26.1% of these patients were using antidepressant medication.^27^ In another Brazilian cross‐sectional study, Fontenelle et al compared patients with blepharospasm and HFS, finding similar rates of psychiatric disorders in both groups, also based on DSM‐IV criteria. Among HFS patients, the prevalence of major depressive disorder was 29% and generalized anxiety disorder 12.9%, while in blepharospasm patients, the rates were 27.3% and 13.6%, respectively. The mean BDI score in HFS patients was 9.7 ± 8.6, which was very similar to that of blepharospasm patients (9.6 ± 7.5, *P* = 0.88).^28^

Lin et al conducted a large cross‐sectional study in China to evaluate patients with HFS. Participants were recruited from 15 movement disorder clinics, and data were collected through questionnaires and medical records. The study included 1003 individuals with HFS, and the findings indicated relatively low rates of psychiatric disorders: 9.1% of patients had depression and 5.7% had anxiety, as measured by the self‐administered Self‐Rating Depression Scale (SDS) and Self‐Rating Anxiety Scale (SAS). The authors also observed that spasms were commonly aggravated by stress, anxiety, talking, exposure to cold wind, and light, while relaxation and facial massage often provided relief. The most frequent associated symptoms were social embarrassment (56%), visual disturbance (52.7%), facial discomfort or pain (41.3%), and sleep disorders (35.2%).^29^

Another cross‐sectional study performed in China aim to evaluate social anxiety among 151 patients with HFS using the Social Avoidance and Distress Scale (SADS). This instrument measures both avoidance and distress, with higher scores reflecting greater severity of social anxiety. The mean total SADS score was 19.07 ± 7.40, with mean subscores of 9.25 ± 3.81 for distress and 9.81 ± 3.71 for avoidance. Considering that total SADS score ≥ 12 indicate high anxiety levels, these findings suggest a clinically significant degree of social anxiety in this population.^30^

Yuksel et al conducted a prospective cohort study in Turkey involving 40 patients with HFS. Participants were evaluated in person for quality of life (using the SF‐36 and HFS‐7 scales) and depressive symptoms (using the BDI) immediately before their routine BoNT injections, and 4 weeks later with the same instruments. The mean BDI scores decreased from 9.28 ± 7.90 before BoNT treatment to 6.70 ± 7.55 after treatment (*P* < 0.01). The proportion of patients with depression (BDI ≥17) also declined from 17.5% (n = 7) at baseline to 12.5% (n = 5) after BoNT injections (*P* < 0.001). Overall, BDI scores improved significantly following BoNT administration.^31^

Wang et al also conducted a prospective cohort study including 95 patients with hemifacial spasm (HFS), who were evaluated before and 2 months after botulinum toxin. Patients were also compared at baseline with 95 age‐ and sex‐matched healthy controls. The study aimed to determine the prevalence of psychological distress in HFS and to evaluate improvements following treatment, as measured by the Symptom Checklist‐90 (SCL‐90). At baseline, age, sex, and average SCL‐90 scores did not differ between HFS patients and controls (*P* > 0.05). Mean scores for interpersonal sensitivity, phobia, anxiety, depression, and somatization were significantly higher among HFS patients than controls (*P* < 0.05 for all). These domains improved significantly after botulinum toxin treatment (*P* < 0.05 for all). At 2 months, 61.3% of HFS patients showed global improvement in psychological distress (*P* = 0.03).^29^

### Sleep

Three studies addressing sleep disorders and the repercussions of HFS on sleep were included in this review. Table 3 summarizes these studies.

**TABLE 3:** Sleep evaluation in HFS patients

Author	Year	Number of patients (n)/Age/Sex	Study type	Main Results
Kim et al^32^	2023	HFS patients: n = 27,106 Age: 55.5 ± 12.7 Female: 17704 (65.3%) Controls: n = 108,424 Age: 55.7 ± 12.6 Female: 70975 (65.5%)	Case–control	The study analyzed nationwide data from South Korea to assess mental illness in patients with HFS. Insomnia was significantly more prevalent in the HFS group (46.2% vs. 13%, *P* < 0.001) when compared to controls (OR: 4.55; 95% CI, 4.16–4.98).
Incirli et al^33^	2019	n = 12 Age: 58 (35–76) Female: 7 (58.3%)	Cross‐sectional	The study evaluated hemifacial spasm during sleep using polysomnography. Polysomnographic study demonstrated arousals between 2 and 11 times due to HFS. Arousals were detected mainly in non‐REM‐II (66%) phases. 52% of the spasms caused an arousal reaction in the EEG.
Kasemsap et al^34^	2015	n = 1 Age: 51 Male	Case report	HFS for 7 years and severe OSA (AHI: 37.3 events/hour). He was also diagnosed with OSA‐induced hypertension. After 1 month of CPAP therapy, he reported that his HFS symptoms had disappeared, and his blood pressure was also under control.

Abbreviations: AHI, apnea‐hypopnea index; CPAP, continuous positive airway pressure; OSA, obstructive sleep apnea.

Kim et al conducted a large population‐based case–control study in South Korea using information from the National Health Insurance Service (NHIS) claims database, covering the period from January 2010 to December 2020. The study included 27,106 HFS patients and 108,424 age‐ and sex‐matched controls. The authors highlighted the clinical relevance of sleep disruption in HFS, reporting that 46.2% of patients met the diagnostic criteria for insomnia based on the International Classification of Diseases, 10th edition (ICD‐10). This prevalence was markedly higher than the 13% observed in healthy controls (*P* < 0.001).^32^

Icirli et al conducted a cross‐sectional polysomnographic study demonstrating that, although the frequency of spasms decreases during sleep, involuntary facial movements persist and contribute to sleep fragmentation. Notably, 52% of spasms were associated with arousal events on EEG. Polysomnography revealed an average of 5.9 ± 2.8 arousals per night caused by abnormal facial movements, occurring predominantly during non‐REM stage 2 (66%), followed by non‐REM stage 1 (20%) and REM sleep (4%). Electromyographic recordings confirmed that while spasm frequency significantly decreased during sleep compared to wakefulness (0.98 ± 0.7 vs. 2.1 ± 1.0; *P* < 0.001), motor activity persisted across all sleep stages, showing a gradual decline throughout non‐REM sleep.^33^

In addition to insomnia, other sleep disorders such as obstructive sleep apnea (OSA) may play a role in the clinical presentation of HFS, as illustrated by the case reported by Kasemsap et al, in which a 51‐year‐old man with severe OSA (apnea–hypopnea index of 37.3 events/hour) also presented with hemifacial spasm and OSA‐induced hypertension. Remarkably, after just 1 month of continuous positive airway pressure (CPAP) therapy, the patient not only achieved better blood pressure control but also reported complete resolution of his HFS symptoms.^34^

### Synthesis of Evidence on Non‐motor Symptoms in HFS

Headache and facial pain appear to be frequent but underrecognized comorbidities among patients with HFS. In the general population, the global prevalence of active headache disorders is approximately 52%, with TTH affecting about 26% and migraine 14% of adults.^35^ In contrast, studies focusing on HFS have reported frequencies of 33 to 60% of pain symptoms, suggesting that these manifestations exceed what would be expected by chance alone in certain populations. For instance, Peeraully et al found that 60% of HFS patients experienced headaches, with 28% reporting onset or worsening precipitated by facial spasms.^18^ Similarly, Harrison et al reported that 24.2% of patients with HFS suffered from headaches and 33% from facial pain,^16^ while Wehrlin et al noted facial pain in 25% of individuals with post‐paralytic spasms.^36^ Collectively, these data support that pain, whether cephalic or periorbital, is frequent in HFS populations.^36^

Peeraully et al also demonstrated that patients with pain had higher motor severity and greater headache‐related disability, as reflected by elevated HIT‐6 scores. HFS severity emerged as the only independent predictor of headache occurrence, emphasizing a dose–response relationship between motor hyperactivity and pain. Notably, 75% of patients with HFS‐related headaches improved after BoNT therapy, whereas none of those with unrelated headaches did, supporting a bidirectional interplay between pain and HFS.^18^ Mizuma et al reported significant reductions in headache intensity and HIT‐6 scores after BoNT injection, with median pain scores decreasing from 7 to 0 (*P* < 0.01) and HIT‐6 improving from 55 to 44 (*P* < 0.001).^15^ These findings parallel those of Harrison et al, who observed improvement in 50% of patients with headache and 82.8% with eye pain after treatment.^16^ Such data highlight the dual role of BoNT in both motor and non‐motor symptom relief.

Several mechanisms may explain the association between headache and HFS. Facial spasms can act as a trigger for primary headache syndromes, as repetitive contractions of periorbital and facial muscles may activate adjacent trigeminal afferents, increasing nociceptive input and sensitization within the trigeminal nucleus caudalis.^37^ This convergence of sensory and motor signals can facilitate temporal and spatial summation, heightening excitability of the trigeminofacial network. Over time, persistent activation may promote central sensitization, resembling mechanisms observed in chronic migraine and tension‐type headache.^15^

**Figure 2:**
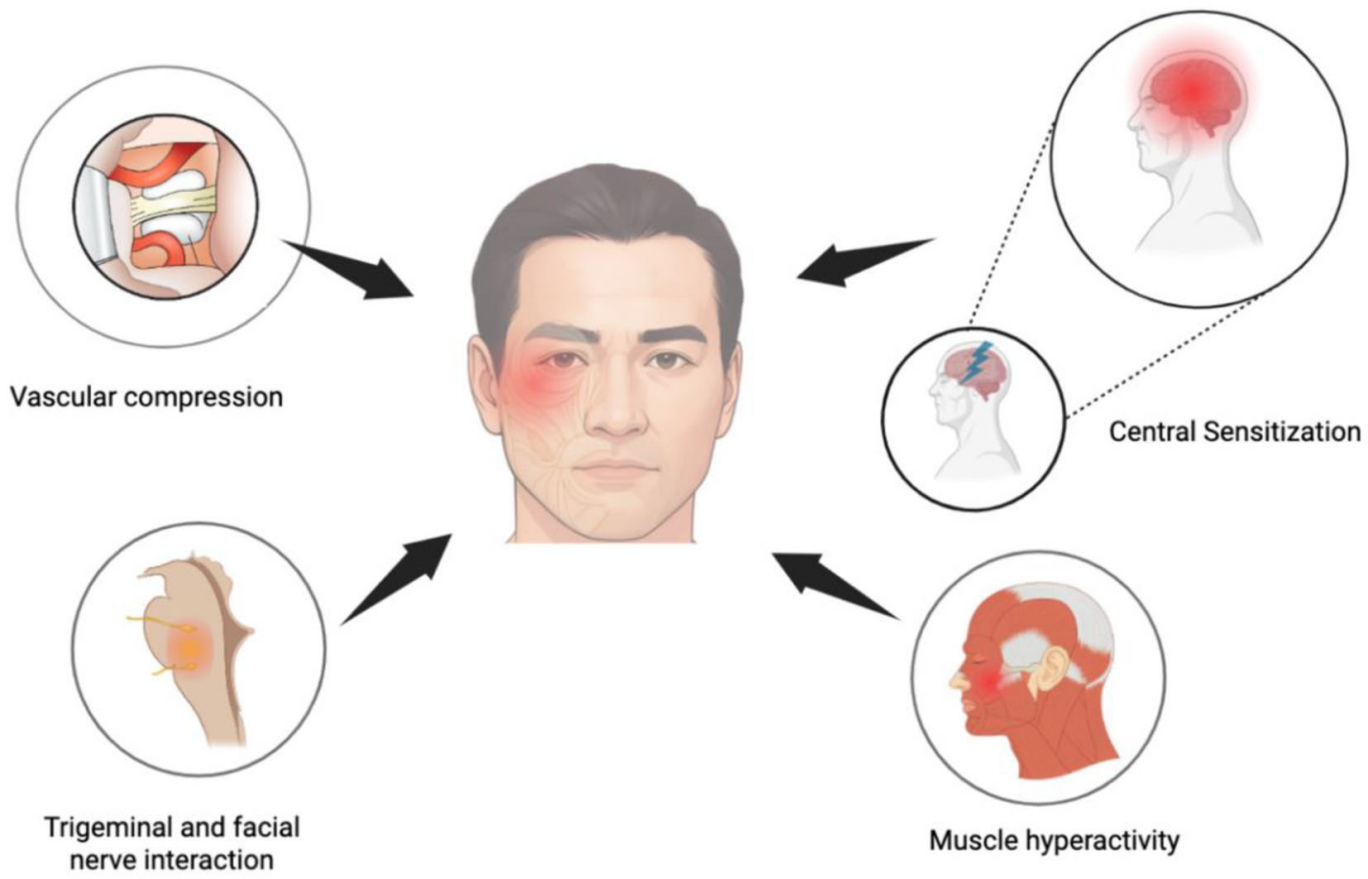
Hypothetical model summarizing proposed mechanisms implicated in headache and facial pain in hemifacial spasm. Created in BioRender. Created by Soares, M. (2025) https://BioRender.com/wbktkgv.

Vascular compression of the facial nerve by vertebrobasilar arteries, may also affect trigeminal pathways, particularly during migraine attacks.^38^ This supports a bidirectional relationship between migraine and HFS. Migraine‐induced trigeminovascular dilation can increase neurovascular contact with the facial nerve, while peripheral irritation from HFS may activate trigeminal nociceptive circuits, triggering migraine.^24^ Clinical reports describing concurrent improvement of both migraine and HFS with topiramate, together with the analgesic effects of botulinum toxin, shown by Wehrlin et al to significantly reduce facial pain (*P* = 0.0001), reinforce its modulatory role in nociceptive processing.^36^ By inhibiting the release of substance P, CGRP, and glutamate, BoNT further supports a shared pathophysiological substrate involving trigeminofacial convergence.^7^ Collectively, these findings indicate that headache and facial pain in HFS arise from intertwined mechanisms of neurovascular compression, central sensitization, and peripheral hyperexcitability, further modulated by psychological factors. Pain in HFS, therefore, probably represents not a coincidental comorbidity but a manifestation of overlapping neural circuits. Figure 2 illustrates a hypothetical conceptual framework summarizing proposed mechanisms of headache and facial pain in HFS.

Depression and anxiety disorders are prevalent non‐motor comorbidities in HFS, with reported prevalences ranging from 9–29% to 5–20%, respectively, consistently higher than in the general population, where lifetime rates are 4–10% for depression and 7–12% for anxiety.^3^, ^26^, ^31^ Tan et al found depression in 16.7% of patients, particularly younger women, and anxiety disorders in 19.5% versus 3.8% of controls (*P* < 0.0001).^25^ Brazilian studies showed comparable findings: Dias et al reported major depression in 13.7% and generalized anxiety in 10.3%,^27^ while Fontenelle et al observed 29% and 12.9%, respectively.^28^ A large Chinese cohort (n = 1003) reported lower rates of depression (9.1%) and anxiety (5.7%), which may reflect the influence of ethnic and cultural factors on symptom expression and reporting.^6^ Despite methodological heterogeneity, these studies consistently demonstrate that emotional distress and psychosocial impairment, often related to the visible and stigmatizing nature of facial spasms, are frequent and impactful features of HFS.

Evidence indicates that psychiatric symptoms and HFS are interrelated, with exacerbation of one often paralleling the other and both improving after BoNT therapy. In a prospective study of 40 patients, Yuksel et al reported a decrease in mean BDI scores from 9.28 to 6.70 (*P* < 0.01) and a reduction in clinically relevant depressive symptoms from 17.5% to 12.5% (*P* < 0.001).^31^ Similarly, Wang et al found significant post‐BoNT improvements in anxiety, depression, somatization, and interpersonal sensitivity (*P* < 0.05 for all), with 61.3% of patients showing reduced psychological distress.^39^

Studies comparing the frequency and severity of obsessive‐compulsive symptoms in HFS and blepharospasm found no differences between matched groups using DSM‐IV criteria and the Yale–Brown scale.^40^ This suggests that these psychiatric symptoms may be reactive or secondary rather than primary disorders, potentially related to the facial manifestations of both conditions, which can have substantial psychosocial impact.

Mechanistically, chronic facial spasms may induce persistent social stress and self‐consciousness, predisposing to anxiety and depression.^31^ Conversely, anxiety and stress can intensify HFS via hypervigilance and sympathetic overactivation, consistent with reports of symptom worsening during emotional stress or fatigue in up to 65% of patients.^29^ This interplay highlights how emotional states can modulate motor control, while visible motor dysfunction, in turn, reinforces psychological distress.

Sleep disturbances are a frequent yet underrecognized non‐motor feature of HFS. In a population‐based Korean study involving 27,106 patients and 108,424 controls, insomnia was diagnosed in 46.2% of HFS cases versus 13% of controls (*P* < 0.001), indicating a threefold higher prevalence.^32^ Such disturbances often manifest as fatigue, daytime somnolence, and psychological distress, which may exacerbate motor symptoms. The increased rate of insomnia may be related to a hyperarousal state induced by recurrent spasms during wakefulness that extends into sleep.^33^

Polysomnographic studies confirm that facial spasms persist across all sleep stages, particularly during non‐REM stage 2, leading to arousal‐related fragmentation. Although their frequency decreases during sleep motor activity does not fully cease, impairing restorative sleep.^33^ Additionally, a case report described resolution of both OSA and HFS after 1 month of CPAP therapy, suggesting a shared pathophysiological link through nocturnal hypoxia and motor hyperexcitability.^34^ Overall, the coexistence of HFS and insomnia likely results from reciprocal mechanisms: hyperarousal, persistent motor activity, central sensitization, and anxiety. It forms a self‐perpetuating cycle of poor sleep and motor worsening.^34^ Recognizing and treating sleep disorders, particularly insomnia and OSA, should therefore be an integral part of HFS management.

This review has several limitations that should be acknowledged. The included studies displayed substantial heterogeneity in design, assessment tools, and outcome measures, which limits direct comparison and the ability to perform quantitative synthesis. Different instruments were used to evaluate psychiatric symptoms, pain, and sleep disturbances, leading to variability in diagnostic thresholds and interpretation. Additionally, most available data originated from Asian populations, particularly from Japan, China, and South Korea, especially regarding psychiatric symptoms, which may limit the generalizability of findings to other ethnic and cultural contexts. However, similar psychiatric outcomes were observed across different ethnic groups (Asian and Latin American), reinforcing the consistency of this association.

Given the predominantly observational nature of the evidence, associations between HFS severity and non‐motor symptoms should be interpreted with caution, as directionality cannot be established. Bidirectional relationships and reverse causation are plausible, whereby non‐motor symptoms may both result from and contribute to motor symptom severity, underscoring the need for longitudinal studies.

Although other non‐motor manifestations such as fatigue, cognitive complaints, and autonomic symptoms have been occasionally described in patients with HFS, the available literature addressing these domains remains limited, heterogeneous, and largely descriptive, with most studies lacking standardized assessments or validated outcome measures, thereby precluding meaningful synthesis. Consequently, the present review focused on pain, psychiatric symptoms, and sleep disturbances, which represent the most consistently reported and clinically relevant non‐motor domains; this selective focus should therefore be considered a limitation. In addition, although several observational studies and case reports were rated as low risk of bias using design‐specific JBI criteria, these ratings must be interpreted in light of inherent methodological constraints, including residual confounding, selection bias, lack of comparators, and limited generalizability. Accordingly, findings derived predominantly from these study types were interpreted as exploratory or hypothesis‐generating, underscoring the need for future studies employing comprehensive, standardized non‐motor assessments and larger, controlled designs in HFS.

Because this review focused on primary HFS, the findings may not be generalizable to secondary forms, in which non‐motor symptoms may be influenced by distinct underlying etiologies. Future studies are needed to clarify non‐motor manifestations in secondary HFS.

Despite current limitations, this work represents one of the first systematic efforts to comprehensively characterize non‐motor symptoms in hemifacial spasm (HFS), integrating data on pain, psychiatric manifestations, and sleep disturbances. By combining clinical and therapeutic perspectives, it offers an updated overview of symptom evolution and treatment response, particularly to BoNT. The emphasis on validated instruments and clearly defined outcomes contributes to a structured understanding of the multidimensional burden of HFS. This integrative framework provides a foundation for future research exploring underlying mechanisms and personalized management approaches. Studies incorporating neuroimaging, neurophysiology, and longitudinal designs are warranted to elucidate central network alterations and optimize multimodal therapeutic strategies.

## Conclusion

Contrary to what is commonly thought, HFS is not limited to motor symptoms, but is frequently accompanied by non‐motor comorbidities such as facial pain, headache, depression, anxiety disorders and sleep disturbances. These factors significantly impair quality of life, with younger patients and those with higher levels of psychiatric symptoms being particularly vulnerable. BoNT remains the mainstay of treatment, effectively reducing motor symptoms and also improving specific non‐motor manifestations, including facial pain and headache.

Given the high burden of psychiatric, sensory, and sleep disturbances, optimal management of HFS should combine BoNT with broader multidisciplinary care. Screening for mood disorders, pain syndromes and sleep problems, alongside behavioral approaches and collaboration across neurology, psychiatry, and sleep medicine, may provide more comprehensive and individualized treatment.

## Author Roles

(1) Research project: A. Conception, B. Organization, C. Execution; (2) Statistical Analysis: A. Design, B. Execution, C. Review and Critique; (3) Manuscript Preparation: A. Writing of the first draft, B. Review and Critique;

M.C.S.: 1A, 1B, 1C, 2A, 2B, 2C, 3A.

J.B.P.: 1B, 1C, 2A, 2B, 2C, 3B.

P.A.S.R.F.: 1A, 1B, 1C, 2A, 2B, 2C, 3B.

## Disclosures

**Ethical Compliance Statements:** We confirm that the approval of an institutional review board was not required for this work as only secondary data were used, no review board was needed. The systematic review was conducted in accordance to PRISMA guidelines. The study followed Helsinki. We confirm that we have read the Journal's position on issues involved in ethical publication and affirm that this work is consistent with those guidelines. Informed patient consent was not necessary for this work.

**Funding Sources and Conflicts of Interest:** This study was financed in part by the Coordenação de Aperfeiçoamento de Pessoal de Nível Superior‐Brasil (CAPES) – Finance code 001. The authors declare that there are no conflicts of interest relevant to this work.

**Financial Disclosures for the Previous 12 Months:** The authors declare that there are no additional disclosures to report.

## Financial Disclosures and Conflicts of Interest

Author disclosures are available in the Supporting Information.

## Supporting information


**TABLE S1.** Risk of bias assessment of the included studies

**Data S1.** Coi_Disclosure.

## Data Availability

Data sharing not applicable to this article as no datasets were generated or analyzed during the current study.
