# Supplementary material for: The Hidden Burden of Hemifacial Spasm: A Systematic Review of Non‐Motor Symptoms
Source: Mov Disord Clin Pract. 2026 Mar 5:10.1002/mdc3.70574. Online ahead of print. doi: 10.1002/mdc3.70574 (PMC13339098; doi:10.1002/mdc3.70574)
Supplement: Supplementary file 1 — TABLE S1. Risk of bias assessment of the included studies [file MDC3-9999-0-s001.docx]

**Supplemental table 1.** Risk of bias assessment of the included studies

| **Author** | **Year** | **Study Design** | **Appraisal Criteria Met (Yes/Total)** | **Overall Risk of Bias*** | **Judgment** |
| --- | --- | --- | --- | --- | --- |
| Punj *et al.* | 2023 | Case report | 7/8 | Low bias risk | High-quality |
| Nakazato *et al.* | 2006 | Case report | 6/8 | Low bias risk | High-quality |
| Iida *et al.* | 2004 | Case report | 4/8 | Moderate risk | Medium-quality |
| Husid *et al.* | 2004 | Case report | 7/8 | Low bias risk | High-quality |
| Fenech *et al.* | 2017 | Case report | 8/8 | Low bias risk | High-quality |
| Cuadrado *et al.* | 2008 | Case report | 5/8 | Moderate risk | Medium-quality |
| Alonso-Navarro *et al.* | 2007 | Case report | 7/8 | Low bias risk | High-quality |
| Kasemsap *et al.* | 2017 | Case report | 8/8 | Low bias risk | High-quality |
| Barahona-Hernando *et al.* | 2012 | Case series | 7/10 | Low bias risk | High-quality |
| Mizuma *et al.* | 2017 | Retrospective cohort | 9/11 | Low bias risk | High-quality |
| Harrison *et al.* | 2008 | Retrospective cohort | 6/11 | Moderate risk | Medium-quality |
| Wang *et al.* | 2022 | Prospective cohort | 9/11 | Low bias risk | High-quality |
| Yuksel *et al.* | 2017 | Prospective cohort | 9/11 | Low bias risk | High-quality |
| Tan *et al.* | 2006 | Case-control | 10/10 | Low bias risk | High-quality |
| Kim *et al.* | 2023 | Case-control | 10/10 | Low bias risk | High-quality |
| Peeraully *et al.* | 2013 | Cross-sectional | 6/8 | Low bias risk | High-quality |
| Lin *et al.* | 2014 | Cross-sectional | 8/8 | Low bias risk | High-quality |
| Fontenelle *et al.* | 2011 | Cross-sectional | 6/8 | Low bias risk | High-quality |
| Dias *et al.* | 2010 | Cross-sectional | 6/8 | Low bias risk | High-quality |
| Tan *et al.* | 2005 | Cross-sectional | 8/8 | Low bias risk | High-quality |
| Cai *et al.* | 2024 | Cross-sectional | 6/8 | Low bias risk | High-quality |
| Incirli *et al.* | 2019 | Cross-sectional | 6/8 | Low bias risk | High-quality |

* Risk of bias was assessed using the JBI Critical Appraisal tools specific to each study design.

† Studies with JBI scores above 70% were categorized as high-quality, those scoring between 50% and 69% as medium-quality, and those scoring 49% or below as low-quality.
